# A case of acute lymphocytic gastritis related to treatment with pembrolizumab for metastatic urothelial carcinoma

**DOI:** 10.1002/iju5.12568

**Published:** 2022-12-20

**Authors:** Yousuke Fukiishi, Hideo Fukuhara, Yoshitaka Kurano, Hiroki Shugimoto, Erika Yamashita, Takashi Karasima, Keiji Inoue

**Affiliations:** ^1^ Department of Urology National Hospital Organization Kochi National Hospital Kochi Japan; ^2^ Department of Urology Kochi Medical School Kochi Japan

**Keywords:** acute lymphocytic gastritis, metastatic urothelial carcinoma, pembrolizumab

## Abstract

**Introduction:**

Immune checkpoint inhibitors such as programmed cell death/−ligand 1 inhibitor and cytotoxic T‐lymphocyte‐associated antigen‐4 inhibitors have been widely used for various advanced malignancies. The mechanism of action for these inhibitors is the improvement of antitumor immunity via T‐cell modulation. On the contrary, immune‐related adverse events such as autoimmune colitis might arise in association with T‐cell activation. Upper gastrointestinal adverse events related to pembrolizumab have rarely been reported.

**Case presentation:**

A 72‐year‐old man underwent laparoscopic radical cystectomy for muscle‐invasive bladder cancer (pT2N0M0). Multiple lymph node metastases appeared in the paraaortic region. First‐line chemotherapy comprising gemcitabine and carboplatin failed to stop disease progression. After the administration of pembrolizumab as second‐line treatment, the patient showed symptomatic gastroesophageal reflux disease. Esophagogastroduodenoscopic biopsy of the gastric body showed severe lymphoplasmacytic and neutrophilic infiltration.

**Conclusion:**

We present acute gastritis related to pembrolizumab. Early eradication therapy may be able to control immune checkpoint inhibitor‐related gastritis.

**Abbreviations & Acronyms**
CMV: cytomegalovirus
CR: complete response
CT: computed tomography
CTLA‐4: cytotoxic T‐cell‐associated antigen‐4
EDG: esophagogastroduodenoscopy
FDG: fluorodeoxyglucose
GI: gastrointestinal
HP: Helicobacter pylori
ICI: immune checkpoint inhibitor
irAE: immune‐related adverse events
MRI: magnetic resonance imaging
ND: not detectable
PD‐1: programmed cell death‐1
PD‐L1: programmed cell death ligand‐1
PLS: prednisolone
PET: positron emission tomography
PPI: proton pump inhibitor

**Keynote message**

Pembrolizumab is used as a second‐line chemotherapy for metastatic urothelial carcinoma. Gastritis related to pembrolizumab is considered rare. We present herein a case of acute lymphocytic gastritis related to treatment with pembrolizumab for metastatic urothelial carcinoma.

## Introduction

ICIs such as PD‐1 and PD‐L1 inhibitors and CTLA‐4 inhibitors have been widely used for multiple solid tumors. Although diarrhea and colitis are the most commonly reported GI adverse events, gastritis related to pembrolizumab is considered rare. We present herein a case of acute lymphocytic gastritis related to treatment with pembrolizumab for metastatic bladder cancer.

## Case presentation

**Fig. 1:**
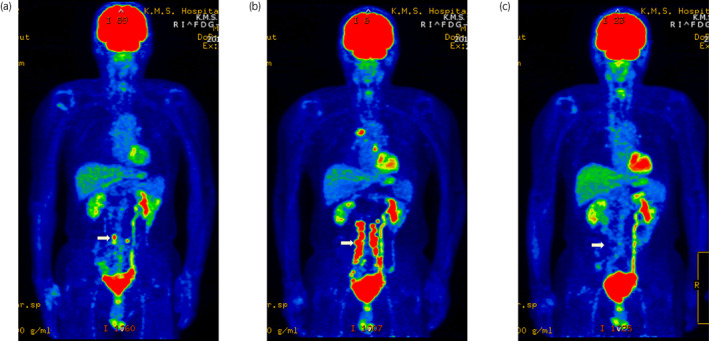
Imaging findings from PET‐CT. PET‐CT of metastatic lesions in abdominal lymph nodes. (a) Abdominal lymph node metastases after surgery (arrow). (b) Progressive disease after two cycles of gemcitabine and carboplatin chemotherapy (arrow). (c) CR after 3 cycles of pembrolizumab (arrow).

A 72‐year‐old man visited our hospital complaining of gross hematuria. There were no GI illnesses in his medical history. Cystoscopy revealed multiple bladder tumors. CT and MRI showed stage cT1N0M0 disease. The patient underwent transurethral resection of the bladder tumors. Complete resection of the bladder tumors was not achievable because of the extensive lesions. The pathological result was high‐grade pT1 urothelial carcinoma. After pathological diagnosis, the patient was treated with two cycles of a gemcitabine and cisplatin regimen as neoadjuvant chemotherapy. The patient then underwent laparoscopic radical cystectomy with the creation of a U‐shaped ileal neobladder and limited dissection of the lymph node. Pathological examination showed high‐grade pT2 urothelial carcinoma with negative resection margins and pN0 (two lymph nodes). Recurrence evaluation after surgery was determined by FDG‐PET‐CT due to reduced renal function. Three months after surgery, FDG‐PET‐CT taken to evaluate the effect of initial postoperative treatment revealed a new appearance of abdominal lymph node metastasis (Fig. 1a). Due to reduced renal function, combination chemotherapy with gemcitabine and carboplatin was administrated. However, enlargement of lymph node metastases was identified on FDG‐PET‐CT after two cycles (Fig. 1b). The patient began treatment with pembrolizumab (200 mg/body administrated every 3 weeks) as second‐line treatment. FDG‐PET‐CT after three cycles of pembrolizumab showed a marked response with the disappearance of FDG accumulation in all metastatic lesions (Fig. 1c).

**Fig. 2:**
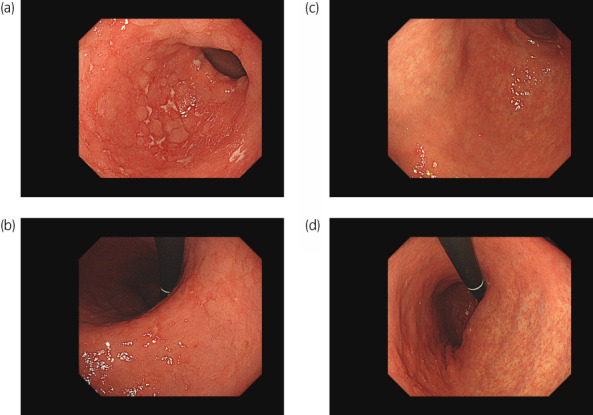
Findings from EDG. (a) Erythematous and edematous gastric mucosa with a whitish membrane. (b) Diffuse erosions in the gastric antrum. Edematous change (c) and erosion (d) in gastric mucosa improved after eradication therapy.

The patient had no adverse effects, but after 10 months complained of anorexia and upper abdominal pain. EDG demonstrated diffusely erythematous and edematous gastric mucosa covered with a whitish, fibrin‐like membrane (Fig. 2a). In addition, diffuse erosions were found in the gastric antrum (Fig. 2b).

**Fig. 3:**
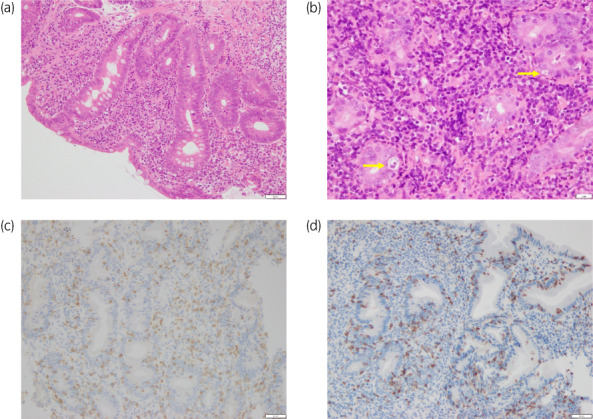
Pathological findings in the gastric epithelium. (a) Infiltration of lymphocytes and plasma cells into the lamina propria. (b) Apoptotic bodies (arrow). (c) Infiltration T cells into epithelium are positive for CD3. (d) Infiltration T cells into epithelium are positive for CD8.

Biopsy specimens revealed inflammatory cell infiltration and apoptosis in the epithelium. High numbers of lymphocytes and plasma cells were observed infiltrating into the lamina propria (Fig. 3a). In addition, T cell infiltration and apoptotic bodies were observed in the gastric epithelium (Fig. 3b). Immunostaining identified these lymphocytes as CD3^+^ (Fig. 3c) and CD8^+^ T‐cells (Fig. 3d) in the epithelium. No histological or immunohistochemical evidence of Helicobacter pylori or cytomegalovirus was apparent. However, the serum H. pylori antibody concentration was elevated (15 U/mL; normal <10 U/mL). The clinical and pathological findings were comparable with lymphocytic gastritis induced by pembrolizumab. The patient received eradication therapy combined with the administration of a PPI, amoxicillin, and clarithromycin for 1 week. Eradication therapy and cessation of pembrolizumab led to improvement of clinical symptoms and findings on EDG without steroid therapy in 4 months (Fig. 2c,d). The patient has since resumed and continued pembrolizumab administration while maintaining CR for 28 months to date.

## Discussion

GI disorders are frequently observed as irAEs following ICI administration, but occur less frequently with PD‐1 inhibitors than with anti‐CTLA‐4 inhibitors, and are almost always lower GI disorders.^1^ CTLA‐4 inhibitors are known to cause early CD4^+^ T cell activation in the lymph nodes, whereas PD‐1 inhibitors target late‐stage T‐cell proliferation, thus facilitating more localized immune responses in the tumor microenvironment.^2^

In malignant melanoma patients treated with pembrolizumab (KEYNOTE‐006 trial), colitis occurs in 2.7% (15/555) of any grade and 1.9% (11/555) of grades 3–5. However, upper GI disorders have only been reported in a few case reports and original papers.^3^

Past case reports have included 17 cases of PD‐1 inhibitor‐induced gastritis. Most patients developed gastritis within 1 year of treatment, but five patients developed gastritis after being treated for longer than 1 year. Twelve patients were treated with steroids. Diarrhea was the most common symptom of colitis, while the main symptoms of gastritis were diarrhea, anorexia, nausea, vomiting, and stomach pain (Table 1).

**TABLE 1:** Reported cases of gastritis induced by the anti‐PD‐1 antibody

No.	Author	Age	Sex	Type of tumor	Drug	Duration of therapy	Symptom	Treatment	CMV	HP	Effect
1	Vandepapelière J	67	F	Melanoma	Pembrolizumab	5 year	Anorexia	PSL	ND	−	Disappeared after 2 months
2	Niahimura Y	68	F	Lung cancer	Pembrolizumab	25 cycles	Anorexia, nausea	PSL	−	−	Disappeared
3	Saito K	71	M	Lung cancer	Pembrolizumab	19 cycles	Epigastric pain	Pembrolizumab withdrawal	ND	−	Disappeared in a week
4	Irshaid L	83	M	Melanoma	Nivolumab	8 cycles	Anorexia, vomiting	PSL	ND	−	Disappeared
5	Sugawara T	73	M	Urothelial carcinoma	Pembrolizumab	16 cycles	Anorexia, Epigastric pain	Pembrolizumab withdrawal	ND	−	Disappeared after 2 months
6	Sugiyama Y	44	M	Lung cancer	Pembrolizumab	3 cycles	Nausea, vomiting	PSL	ND	ND	Disappeared
7	Boike J	93	F	Lymphoma	Nivolumab	6 months	Dysphagia	PSL	−	−	Disappeared
8	O'Neill C	61	F	Lung cancer	Pembrolizumab	13 cycles	Epigastric pain	PSL	ND	−	Disappeared
9	Omotehara S	57	F	Renal cell carcinoma	Nivolumab	12 cycles	Epigastric pain, anorexia	PSL	−	ND	Disappeared in 2 weeks
10	Nebdi R	44	M	Lung cancer	Pembrolizumab	1 year	Vomiting	PSL	−	−	Disappeared
11	Vindum H	16	F	Melanoma	Nivolumab	6 cycles	Nausea, vomiting	Infliximab	ND	ND	Disappeared
12		75	F	Melanoma	Nivolumab, Ipilimumab	2 months	Epigastric pain, Anorexia	PSL	−	+	Disappeared in 4 weeks
13	Gaffuri P	75	M	Melanoma	Pembrolizumab	8 cycles	None	PSL	−	−	Disappeared in weeks
14	Yip RHL	44	M	Melanoma	Pembrolizumab	1 month	Gastroesophageal reflux disease	ND	ND	ND	ND
15	Kobayashi M	77	M	Lung cancer	Nivolumab	4 months	Epigastric pain	PSL	−	−	Disappeared in a few days
16	Alhatem A	78	M	Lung cancer	Nivolumab	1 year	Anorexia	PSL	−	−	ND
17	Jun Lu	66	F	Colon cancer	Atezolizumab, pembrolizumab	5 cycles	Epigastric pain, nausea, vomiting	Ganciclovir	+	−	ND

Gastritis resulting from ICI use has endoscopic and immunologic features similar to those of gastritis induced by H. pylori, the gross findings are similar, and gastric biopsy is useful for definitive diagnosis.^4^ Three characteristic endoscopic findings are important to diagnose ICI‐induced gastritis: (i) network‐pattern erosion or ulcer in the antrum; (ii) erythematous and edematous mucosa with excessive whitish purulent discharge in the whole stomach; (iii) extremely fragile mucosa.^5^ This present case showed diffuse erosions in the antrum and erythematous and edematous mucosa in the whole stomach. Histologically, diffuse and moderately to severely active chronic gastritis combined with increased intraepithelial lymphocytes and increased apoptotic activity are the most helpful features for diagnosis of ICI‐associated gastritis.^6^ Apoptosis is a most noteworthy histological finding in ICI‐induced gastritis. In the present case, apoptosis appeared in the epithelium. Hayama *et al*. examined immunohistological findings in the gastric mucosa of gastritis caused by ICI use and reported infiltration predominantly comprising CD4 or CD8 lymphocytes, and not regulatory lymphocytes such as Foxp3 lymphocytes, as well as expression of PD‐L1, rather than vascular endothelial growth factor receptor within gastric cells in the mucosa.^7^ These morphological and immunohistological features may offer useful discriminators of ICI‐induced gastritis.

The patient in this case was positive for H. pylori antibodies. Similar gastritis has been reported after the administration of ipilimumab.^8^ Although H. pylori infection may have triggered gastritis in this patient, the diagnosis of GI‐ irAE was made based on pathological findings of irAE reported that anti‐CTLA‐4 antibodies may induce extensive gastritis in H. pylori‐infected patients in an in vivo model of H. pylori infection using mice treated with anti‐CTLA‐4 fragments.^9^ The present patient received eradication therapy and showed improvement after approximately 20 weeks without corticosteroids.

In cases such as this, if the disease is diagnosed early and appropriate eradication therapy is initiated, corticosteroid therapy may be avoidable, and continue to receive pembrolizumab.

## Author contributions

**Hideo Fukuhara:** Conceptualization; writing – review and editing. **Yousuke Fukiishi:** Data curation; writing – original draft. **Yoshitaka Kurano:** Writing – review and editing. **Hiroki Shugimoto:** Writing – review and editing. **Erika Yamashita:** Writing – review and editing. **Takashi Karasima:** Supervision. **Keiji Inoue:** Supervision.

## Conflict of interest

The authors declare no conflicts of interest.

## Approval of the research protocol by an Institutional Reviewer Board

Not applicable.

## Informed consent

Informed consent was obtained from the patient for the publication of this case report and accompanying images.

## Registry and the Registration No. of the study/trial

Not applicable.
